# Additives for vaccine storage to improve thermal stability of adenoviruses from hours to months

**DOI:** 10.1038/ncomms13520

**Published:** 2016-11-30

**Authors:** Maria Pelliccia, Patrizia Andreozzi, Jayson Paulose, Marco D'Alicarnasso, Valeria Cagno, Manuela Donalisio, Andrea Civra, Rebecca M. Broeckel, Nicole Haese, Paulo Jacob Silva, Randy P. Carney, Varpu Marjomäki, Daniel N. Streblow, David Lembo, Francesco Stellacci, Vincenzo Vitelli, Silke Krol

**Affiliations:** 1 European School of Molecular Medicine (SEMM), IFOM-IEO-Campus, via Adamello 16, Milan 20139, Italy; 2 Università degli Studi di Milano, Milan 20122, Italy; 3 Fondazione I.R.C.C.S. Istituto Neurologico Carlo Besta, IFOM-IEO-campus, via Adamello 16, Milan 20139, Italy; 4 IFOM—FIRC Institute of Molecular Oncology, IFOM-IEO-campus, via Adamello 16, Milan 20139, Italy; 5 Instituut-Lorentz for theoretical physics, Leiden University, 271, Niels Bohrweg 2, NL 2333 CA Leiden, The Netherlands; 6 Fondazione CEN—European Centre for Nanomedicine, Piazza Leonardo da Vinci, 32, 20133 Milan, Italy; 7 Laboratory of Molecular Virology and Antiviral Research, Department of Clinical and Biological Sciences, University of Turin, S. Luigi Gonzaga Hospital, Regione Gonzole 10, 10043 Orbassano, Italy; 8 Vaccine & Gene Therapy Institute, Oregon Health & Science University, 505 NW 185th Avenue, Beaverton, Oregon 97006, USA; 9 Institute of Materials and Interfaculty Bioengineering Institute, École polytechnique fédérale de Lausanne, STI IMX SUNMIL MXG 030, Station 12, CH-1015 Lausanne, Switzerland; 10 Department of Biological and Environmental Science/Nanoscience Center, University of Jyväskyla, Survontie 9, 40500 Jyväskyla, Finland; 11 Laboratory of Translational Nanotechnology, I.R.C.C.S. Istituto Tumori Giovanni Paolo II, viale Orazio, Flacco 65, Bari 70124, Italy; 13; ; ;

## Abstract

Up to 80% of the cost of vaccination programmes is due to the cold chain problem (that is, keeping vaccines cold). Inexpensive, biocompatible additives to slow down the degradation of virus particles would address the problem. Here we propose and characterize additives that, already at very low concentrations, improve the storage time of adenovirus type 5. Anionic gold nanoparticles (10^−8^–10^−6^ M) or polyethylene glycol (PEG, molecular weight ∼8,000 Da, 10^−7^–10^−4^ M) increase the half-life of a green fluorescent protein expressing adenovirus from ∼48 h to 21 days at 37 °C (from 7 to >30 days at room temperature). They replicate the known stabilizing effect of sucrose, but at several orders of magnitude lower concentrations. PEG and sucrose maintained immunogenicity *in vivo* for viruses stored for 10 days at 37 °C. To achieve rational design of viral-vaccine stabilizers, our approach is aided by simplified quantitative models based on a single rate-limiting step.

## Abstract (web summary)

Keeping viral vaccines cold from the manufacturers to patients is problematic and costly. Here, Krol and others show additives that can significantly improve at very low concentrations the storage of adenovirus type 5 at ambient and elevated temperature.

## Graphical abstract

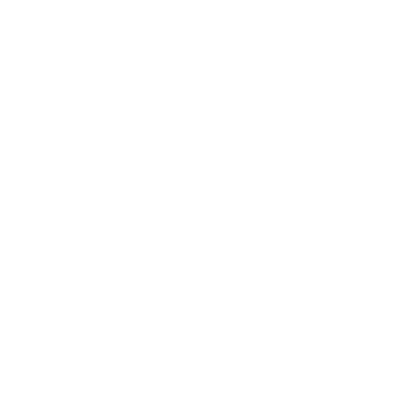

Vaccination saves millions of lives every year. Attenuated, genetically modified or inactivated viruses are still the key active ingredients of many vaccines, despite recent progress in the use of virus-like particles or proteins in vaccine preparations. Maintaining the potency of these viral particles against degradation is a major challenge in providing proper immunization services. Typically, this requires keeping vaccines refrigerated at all times from production to administration—a major undertaking especially in remote regions of developing countries. Termed the cold chain problem, the high cost and risk associated with protecting vaccines from deterioration has been identified by the World Health Organization as one of the most important challenges for the extension of global vaccination programmes^1^. The development of thermally stable formulations for the major vaccines could relieve bottlenecks in the vaccine supply chain^2^. The addition of additives to existing vaccines usually means to get again through the costly process of approval by health authorities, which is commercially unattractive^3^. Therefore, proposing solutions that use inexpensive additives for stabilization increases the likelihood of their adoption by vaccine manufacturers in the development of new vaccines

There are two temperature-induced risk factors for vaccines, aluminium salt aggregation due to freezing (aluminium is a salt adjuvant that serves as an immune booster)^4^ and inactivation of the attenuated or inactivated virus by exposure to elevated temperatures^2^. While the first risk is dealt with stabilizers such as anti-freezing agents^5^,^6^, sucrose (in liquid vaccine preparation at millimolar concentration or in solid vaccines in milligram quantities) being the most common^7^, the reasons for the second one are largely unknown and hence only empirical solutions are available. Few successful approaches to stabilize viral vaccines have been proposed^4^. One strategy is the freeze-dried preparation of vaccines, such as hepatitis B, rotavirus, measles, mumps and rubella virus vaccines^8^. Another approach is to immobilize viral particles in a sugar glass on a filter^9^. Solid preparations are known to drastically improve the stability at elevated temperatures. One approach that has been in development in recent years is the addition of sucrose at molar concentrations to vaccine formulations^4^,^5^,^9^,^10^,^11^,^12^. Another approach uses silk fibres. These silk fibres stabilize the vaccine during transport and storage in its powder form as well as the reconstituted vaccine^13^.

Earlier empirical approaches stabilize liquid formulations of Ad5 viral vaccines were described by Evan *et al*.^10^. They identified freeze-thaw damage, surface adsorption and free radical oxidation as reasons of virus destruction. They tested a combination of buffering molecules, ions and sucrose and found a solution containing 5 w/v% sucrose as the most effective cryoprotectant. Other compounds were polysorbate-80 as non-ionic surfactant, metal-ion catalyzed free-radical oxidation inhibitor, the free-radical oxidation inhibitors, ethanol and histidine, and the metal-ion chelator ethylenediaminetetraacetic acid preserving 99% infectivity for Ad5 viruses stored for 24 months at 4 °C, and 95% for 1 day at 37 °C. Stewart *et al*.^12^ showed that a preservation of infectivity of adenovirus for a sucrose-stabilized liquid formulation for up to 6 weeks at 40 °C, hence confirming the stabilizing effect of sucrose.

Here we propose and evaluate a diverse set of additives to stabilize exemplarily adenoviral vaccines at ambient temperatures. Using *in vitro* and *in vivo* measurements of adenoviral infectivity over time, we demonstrate how vastly different media—simple sugars, long-chain polymers and coated nanoparticles (NPs), with concentrations spanning many orders of magnitude—can be used to enhance the lifetime of viral formulations from several days to weeks or months. We interpret these experimental results in the context of a model of adenoviral capsid degradation that compares the effects of different physical processes that may influence virus lifetimes.

## Results

### Maintenance of adenoviral infectivity *in vitro*

Our additives are tested on genetically modified adenovirus serotype 5 (Ad5). Adenovirus (Ad) is one of the most intensely investigated viral particles for genetically engineered chimeric vaccines. Ad chimeric vaccines^14^ for highly pathogenic viruses are in different stages of development: rabies^15^ is commercially available (for fox and skunk vaccination: ONRAB), Ebola^16^, human immunodeficiency virus^14^ and H5N1 avian flu^17^,^18^ are under development. The virus itself is chosen as a good vehicle because even if it is still infective, it only causes mild self-limiting respiratory syndrome in immune competent individuals. Genetically engineered Ad is especially vulnerable to the stability problem stated above^19^,^20^. For example, it was found that deletion of two gene regions (E1 or E1/3) and replacement by shorter or longer pieces of foreign genes leads to variations in the thermal stability of Ad5 (ref. 21).

To address the problem of Ad stability (and for viruses in general) we chose to work with a genetically modified replication-deficient Ad5 with deleted E1/E3 gene region replaced by an inserted green fluorescent protein (GFP) reporter gene (Ad-GFP). However, while the Ad vector expressing GFP was ideal for optimizing stability protocols using *in vitro* assays and facilitating the read-out of infection, Ad-GFP is not a vaccine. Therefore, we utilized *in vivo* an Ad expressing Chikungunya (CHIKV) non-structural protein 1 (NSP1) for a proof-of-concept experiment, to show that our additives do not interfere with the ability of Ad to elicit immunity.

**Figure 1:**
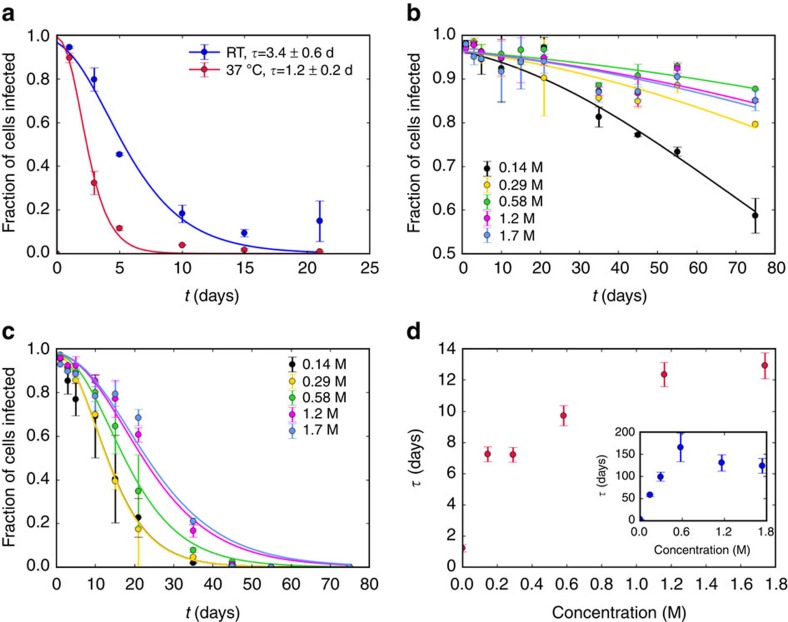
Maintained Ad5 infectivity versus time in PBS and sucrose. (**a**) Fraction of cells infected versus storage time (days) of Ad5 in PBS at RT (blue) and at 37 °C (red). The curves are fits to the expected infectivity given an exponential decay of viable Ad5 particles with characteristic lifetime *τ*, equation (1). (**b**,**c**) Fraction of cells infected versus storage time in different sucrose concentrations ranging from 0.14 to 1.7 M at RT (**b**) and at 37 °C (**c**). (**d**) The lifetime of Ad5 virus as extracted from the fits at 37 °C (main panel) and at RT (inset). (A comparison with the infectivity change for herpes simplex virus-2, another example of double-stranded DNA viruses, can be found in Supplementary Fig. 1.) The experiments were performed in duplicate. Error bars represent the s.d. d, days.

The degradation of Ad5 virions in a given storage medium was evaluated experimentally by measuring the infectivity of the virus preparation over time at two storage temperatures, room temperature at 25 °C (RT) and 37 °C. Infectivity was measured by the expression of the reporter gene GFP, evaluated by fluorescence-activated cells sorting, and quantified as percentage of cells infected. We first established the baseline stability of Ad5 by measuring its infectivity in the standard storage medium of phosphate-buffered saline (PBS) over 21 days at the two storage temperatures. The results are shown in Fig. 1a (symbols).

The infectivity drops to below 20% over the span of a few days; the loss of infectivity is faster at 37 °C than at RT, consistent with loss of infectivity due to thermal degradation of virus particles. The fact that Ad5 viruses are prone to thermally induced changes^11^ was observed in our dynamic light scattering (DLS) measurements, where an increase in hydrodynamic diameter with increasing temperature was determined (Supplementary Fig. 1 (grey line)). The reasons for the change in hydrodynamic diameter can be due to changes of proteins or the capsid and hence a change in the ions and water shell around the capsid. A direct measurement of the fluctuation are too small and too fast to be observed in DLS and in general by other existing techniques.

The simplest model for loss of infectivity assumes that individual virus particles, or virions, degrade from a viable to a disrupted state at a constant rate. This implies an exponential decay in the population *n*(*t*) of infectious virions in a fixed volume of preparation: *n*(*t*)=*n*_0_e^−*t*/*τ*^, where *n*_0_ is the initial population and *τ* is the inverse decay rate, equivalent to the mean lifetime of an individual virion. If *N*_*c*_ cells are inoculated by this preparation, the absorption of viral particles by cells follows a Poisson distribution. The fraction of cells infected equals the probability that a cell absorbs at least one infectious particle:


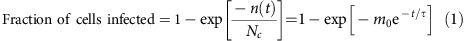


where *m*_0_ is the initial ratio of infectious virions to cells. Whereas *m*_0_ is set by the details of the infectivity assay, the lifetime *τ* completely quantifies the loss of viable virions over time.

To test the hypothesis of constant-rate degradation, we performed fits of the infectivity data to equation (1) with *m*_0_ and *τ* as free parameters (Fig. 1a, solid lines). The model successfully captures the infectivity trend, which is consistent with a constant decay rate of infective virions (quality-of-fit statistics are provided in the Supplementary Discussion). The lifetime *τ* is found to be around 3 days at RT and 1 day at 37 °C in PBS. These lifetime estimates provide the baseline against which the efficacy of the proposed lifetime-prolonging additives is measured.

Exponential decay of virions at a constant rate is characteristic of a single-step degradation reaction. The lifetime (inverse decay rate) for such a reaction takes on the Arrhenius form^22^


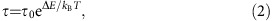


where *τ*_0_ is a relaxation time or inverse attempt frequency characterizing the dynamics of the degradation mechanism, Δ*E* is the energy barrier separating the infectious from the degraded state, *k*_B_ is the Boltzmann constant and *T* is the temperature. The baseline values of the relaxation time and energy barrier in PBS can be estimated from the measured lifetimes at two different temperatures (Fig. 1a). Assuming that Δ*E* and *τ*_0_ are unchanged, the observed change in lifetime with temperature corresponds to an activation energy of Δ*E*≈1 × 10^−19^ J, consistent with previously reported activation energies for the degradation of phage viruses^23^. The corresponding relaxation time is *τ*_0_≈6 × 10^−7^ s.

While equation (2) is completely general for single-step reactions, predicting the effect of storage media on the crucial parameters *τ*_0_ and Δ*E* requires a mechanistic model for virus degradation. A detailed analysis of all the stochastic processes that might lead to loss of infectivity of the Ad5, taking into account its complex structure, is beyond the scope of this work. However, our observation that loss of infectivity is consistent with single-step activation simplifies the search for degradation models, since the degradation rate is primarily determined by a single physical process. In the Supplementary Discussion section, we present a model that assumes that adenovirus degradation occurs due to spontaneous mechanical rupture of the capsid.

To test the adequacy of the infectivity assay and the fitting procedure, we studied the thermal stability of Ad5 solutions in the presence of sucrose, a known effective additive for viral vaccine stabilization (for example, JE-Vax, MMR-II, Pentacel DTaP+IPV+Hib, Rotarix, Varivax and Zostavax)^7^. The results for Ad5 virus stability as a function of sucrose concentration for the first 75 days of the experiment are shown in Fig. 1b–d. At RT, the virus lifetime in 0.3 M sucrose solution is 30-fold higher than that in PBS. Lifetime estimates at higher concentrations proved unreliable because the measured fall in infectivity after 75 days was <20% for the chosen assay. At 37 °C, an 8-fold extension of the lifetime relative to PBS is obtained in 0.6 M sucrose, but the effect saturates at higher concentrations.

These observations confirm earlier findings by other researchers that observed an increased stability for either Ad5 viruses or other viruses at higher sucrose concentration^4^,^11^,^24^,^25^,^26^. In the Supplementary Information, we show that sucrose was also able to stabilize herpes simplex virus-2, another example of double-stranded DNA virus (Supplementary Fig. 1A,B) or vesicular stomatitis virus (VSV), a RNA virus (Supplementary Fig. 1C). The effect on VSV is not significant for the stabilization as the RNA virus *per se* is more stable in contrast to the double-stranded DNA viruses.

The effect of sugars as stabilizers against protein unfolding is known^5^,^25^,^26^,^27^,^28^,^29^. Because of this effect sucrose has been used as cryo-stabilizer for freeze-dried vaccines. Yet, it has been shown that sucrose-induced stabilization of the viral capsid proteins alone does not necessarily lead to virus capsid stabilization^25^. More importantly, the protein stabilization effect is broadly observed with many sugars^9^,^24^,^30^,^31^, but only sucrose and trehalose seem to be effective in stabilizing viral capsids^4^,^9^.

In this study, we tested other organic compounds such as glycerol, glucose, maltose and raffinose in the same concentration range and with approximately the same viscosity at RT. Weak stabilization was often observed at lower concentrations, but at higher concentration the effect was smaller or in some cases destabilizing (representative data sets for different saccharides and glycerol at different concentrations after storage of Ad5 for 5 days at 37 °C are shown for sucrose on VSV in Supplementary Figs 1C and 2). We usually observed that di- and tri-saccharides usually stabilize the virus capsid better than monosaccharides. However, we observed that glycerol is clearly destabilizing (Ad5 and VSV).

Taken together, our results show that neither a general stabilizing effect against protein unfolding nor an increase in medium viscosity, both of which are common to many sugars, is sufficient to explain the enhancement of adenovirus lifetime due to sucrose. In the Supplementary Discussion section, we explore one possible alternative in the context of our model of mechanical adenovirus degradation: if the loss of infectivity happens via spontaneous rupture of the capsid due to thermal fluctuations and the mechanical stresses exerted by the confined genome, it could be mitigated by an enhancement of capsid protein–protein-binding strengths due to sucrose. A crude model of this process shows that a small increase of 1% in the binding strength of the capsid protein–protein interactions would be sufficient to explain the marked stabilization of Ad5 at both temperatures (Supplementary Discussion section). Since sucrose is known to enhance protein–protein binding at molar concentrations by modifying protein hydration properties^26^, our hypothetical mechanism provides an interesting avenue for future studies. However, we stress that spontaneous rupture of the capsid is just one possible degradation mechanism that is consistent with the observed trends.

Next, we focused on finding additives that may be effective at lower concentrations. Motivated by the possibility of protein hydration playing a role in stability, we studied the effect of high-molecular-weight polyethylene glycol (PEG), which is known to affect protein–protein interactions by changing their hydration^32^. Covalent binding of PEG was used in previous studies by other researchers to prevent recognition by inhibiting Ad5 antibodies^33^, as well as stabilize the virus under harsh conditions^34^. One approach to increase the stability was the genetic engineering of viral vectors or chemical modification such as crosslinking of capsid proteins^35^,^36^,^37^. These approaches include also the interaction with nanomaterials and organic polymeric structures^35^ as well as a predictive model^36^.

**Figure 2:**
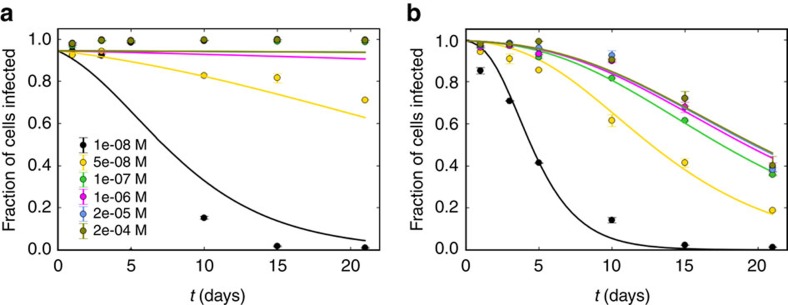
Maintained Ad5 infectivity versus time in PEG8000 solutions. (**a**,**b**) Fraction of cells infected versus storage time for Ad5 stored in the presence of different concentrations of PEG at RT (**a**) and at 37 °C (**b**). Both panels share the same legend. The curves are fits to the expected infectivity given an exponential decay of viable Ad5 particles with time. The experiments were performed in duplicate. Error bars represent the s.d.

The effect of PEG8000 on adenovirus infectivity versus storage time at various PEG concentrations is shown in Fig. 2. In addition, the lifetime at 37 °C versus the concentration was calculated (Supplementary Fig. 3). Surprisingly, we observed stabilization both at RT and at 37 °C even at micromolar concentrations (far lower than the concentration needed for virus precipitation^32^). At RT, no measurable loss of infectivity was observed in our assay for PEG concentrations above 10^−6^ M. At 37 °C, the lifetime reached a plateau at 10 days (Supplementary Fig. 4). Since the effect of PEG on physical properties such as protein solubility^38^, osmotic pressure^39^ and viscosity^40^ is negligible at these low bulk concentrations, it is likely that the capsid attracts PEG molecules via van der Waals forces to locally enhance the PEG concentration in its vicinity. Such a local enhancement, together with the exponential dependence of the lifetime on the activation energy Δ*E* (equation (2)), could explain the large stabilization effect at small PEG concentrations as long as the local PEG concentration is high enough to affect Δ*E*. DLS data indicate that PEG increases the thermal stability of the viral capsid at increasing temperatures up to 40 °C (Supplementary Fig. 1 (yellow line)).

PEG might be the ideal solution for the challenge of viral vaccine stabilization, as it is a Food and Drug Administration-approved polymer. Unfortunately, it does generate production of anti-PEG antibodies or hypersensitivity in up to 50% of people exposed to PEG^41^.

**Figure 3:**
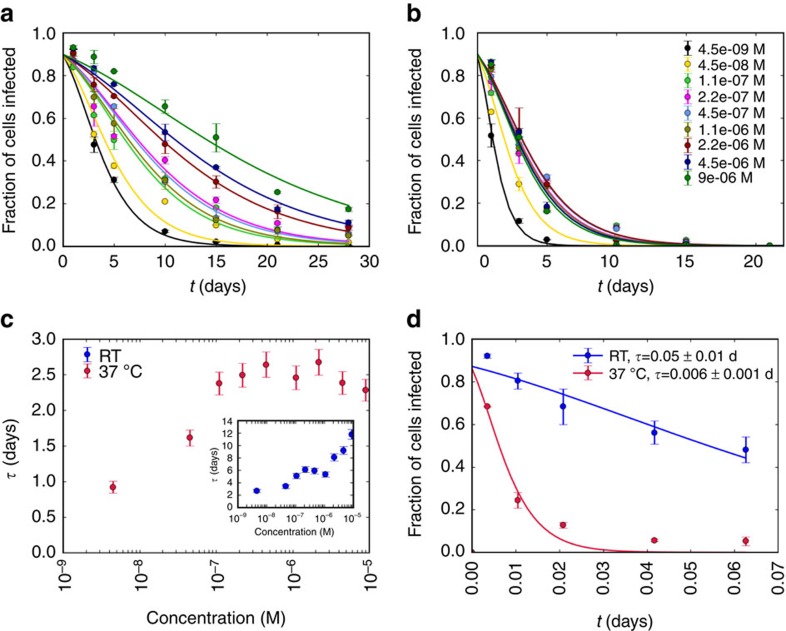
Maintained Ad5 infectivity versus time in the presence of anionic NPs. (**a**,**b**) Fraction of cells infected versus storage time in days for Ad5 stored in presence of different concentrations of anionic MUS:OT NPs at RT (**a**) and at 37 °C (**b**). Curves are fits to the expectation for an exponential decay of viable Ad5 particles with time. (**c**) Characteristic lifetime *τ* extracted from the fits at 37 °C (main panel) and at RT (inset). (**d**) Fraction of cells infected versus storage time in days for Ad5 stored in presence of cationic *N*,*N*,*N*-trimethyl(11-mercaptoundecyl) ammonium chloride:OT NPs at a single concentration of 10^−7^ M in PBS at RT (blue) and at 37 °C (red) along with the fit to the expected loss of infectivity. The experiments were performed in duplicate. Error bars represent the s.d. d, days.

Finally, we tested the effect of electrostatic interactions between the virus and NPs of similar size to PEG (whose radius of gyration is 3.5 nm (ref. 42)) but with a high surface charge density imparted by charged ligands^43^. Anionic gold NPs with core diameter of 2.8±0.7 nm (transmission electron microscopy (TEM) images and particle size distribution are reported in Supplementary Fig. 4) were coated with 11-mercaptoundecansulphonate (MUS) and octanethiol (OT) in a 4:1 stoichiometric ratio as measured by ^1^H-nuclear magnetic resonance, bearing a zeta potential of −35 mV. These NPs were mixed with the virus in the concentration range of 1 × 10^−8^ to 2 × 10^−6^ M (Supplementary for details on molar concentration calculations for these NPs). Virus stability was measured for up to 28 days at RT and at 37 °C (Fig. 3a–c). A gradual but significant increase in virus lifetime with NP concentration was observed, up to 12 days at RT and saturating at 2.5 days at 37 °C. As with PEG, the bulk properties of the solution were practically unchanged at the small values of NP concentration, suggesting a local enhancement of NP concentration near the virus capsid.

In contrast to PEG, however, the enhancement of additive concentration near the virus could be independently verified in the case of NPs. DLS and cryo-TEM measurements (Supplementary Fig. 5) of Ad5 in the presence of NPs support the formation of a cloud of NPs around virus particles as a potential reason for the stabilization of the virus capsid. These observations imply an attractive interaction between Ad5 and NPs. At the colloidal scale, the prominent interactions are van der Waals and screened Coulomb interactions. We estimate the effective pair potential between a NP and a virus particle from these two contributions, as discussed in detail in the Supplementary Discussion section. The NP and virus are both assumed to be uniform spheres. Although the electrostatic component of the interaction is highly repulsive, the van der Waals forces are sufficient to promote an attraction of −1.3 *k*_B_*T* at around 3 nm separation. The low binding strength, by itself, is not sufficient to promote a thermodynamically stable complex of NPs bound to the virus particle. However, Ad5 has a non-uniform distribution of charged domains on its surface, which includes regions that are locally electropositive even though the net surface charge of the virus is negative. The binding energy is expected to be lower (that is, stronger) than −1.3 *k*_B_*T* in these domains, providing a possible mechanism for a thermally stable local enhancement of MUS:OT NP concentration near the virus surface (Supplementary Figs 5 and 6).

A cloud of highly charged macroions changes the capsid environment in two ways. On one hand, the enhanced concentration of neutralizing counter ions applies an osmotic pressure on the capsid due to the Donnan effect^44^. On the other hand, the accompanying negative electrostatic potential makes exposure of the negatively charged DNA energetically unfavourable^45^. Changing the sign of the macroion charge does not affect the first effect but drastically changes the second, by making DNA exposure more favourable and potentially enhancing virus degradation. To help elucidate the mechanism for virus stabilization due to NPs, we repeated our measurements with cationic NPs of similar size, but coated with *N*,*N*,*N*-trimethyl(11-mercaptoundecyl) ammonium chloride and OT in 1:1 ratio resulting in a zeta potential of +60 mV. These positively charged NPs have a core diameter of 4.9±1.1 nm (TEM images and particle size distribution in Supplementary Fig. 7). At a concentration of 10^−7^ M, the cationic NPs show a strong destabilizing effect, reducing the lifetime of virus particles from days to minutes (Fig. 3d). In this case, both, the van der Waals and electrostatic contributions are attractive, indicating a high propensity for NPs to bind to the virus (Supplementary Fig. 8). This observation is consistent with the electrostatic interactions, rather than osmotic pressure changes, being the main determinant of virus stability in the presence of highly charged NPs. We expect anionic NPs to induce a stabilizing effect regardless of their constituents.

To test the generality of this approach, we measured the stabilization of Ad5 at RT and at 37 °C for a number of other anionic gold NPs. These other tested particles varied in size (20 nm citrate gold NPs) or ligand shell composition (ranging from particles coated solely with MUS to NPs coated with mixtures of 3-mercapto-1-propane sulphonic acid and OT). As shown in Supplementary Fig. 9 all tested negatively charged NPs show a stabilization of the viral infectivity at day 5 and 37 °C.

As a first step to establishing the feasibility of our approach for real vaccines, we introduce some initial *in vivo* studies with an Ad expressing the NSP1 (Ad-NSP1) from Chikungunya virus. NSP1 is highly targeted by T cells in mice, monkeys and humans, and is a potential vaccine target^46^,^47^. Because NSP1 is a cytoplasmic protein, the Ad-NSP1 does not elicit neutralizing antibody responses but induces a robust T-cell response. This potential vaccine is being tested in preclinical trials because it elicits a very robust T-cell response by 10 days post vaccination. This vaccine approach is in line with using Ad vector in general as a delivery system for chimeric vaccines (for example, currently ongoing clinical trials for Ad-based Ebola vaccines). Determining whether stabilization of the Ad-NSP1 vaccine enhances immunogenicity after long-term storage will indicate the feasibility and translatability of the additive-based approach to vaccines.

**Figure 4:**
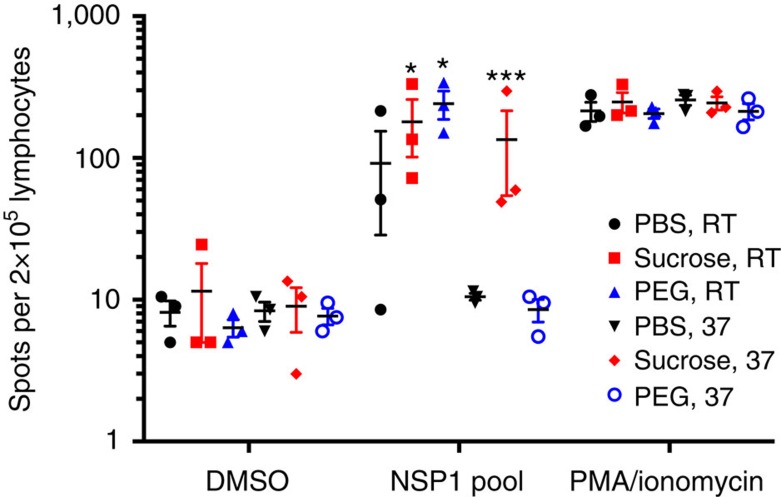
*In vivo* experiments with stabilized Ad-NSP1 after 10 days of incubation in PEG or sucrose solution. Adenovirus vector expressing NSP1 from Chikungunya virus was incubated for 10 days at RT or at 37 °C in PBS, 0.6 M sucrose or 1 μM PEG. Mice were vaccinated by intramuscular injection of 100 μl of Ad-NSP1 from each stabilization condition (*n*=3 per group). At 10 days post vaccination, mouse splenocytes were analysed by interferon gamma ELIspot for the frequency of T cells responding to NSP1 peptides. Ad-NSP1 stabilized in sucrose significantly increased the frequency of T cells responding to NSP1. Photographs of the wells of ELIspot analysis can be found in Supplementary Fig. 8. Data are representative of two independent experiments. Centre values indicate the mean, and error bars represent the s.e.m. DMSO, dimethylsulphoxide; PMA, phorbol myristate acetate.

To determine the impact of long-term stabilization on Ad vector immunogenicity, we incubated Ad-NSP1 either in PBS, 0.6 M sucrose, 0.9 mg ml^−1^ of the anionic MUS:OT NPs or 10^−6^ M PEG8000 for 10 days at RT or at 37 °C. At the end of the incubation period, aliquots of virus from each condition were titred by limiting dilution on 293iq cells. The sucrose solution preserved Ad-NSP1 infectivity >2.1-fold over PBS alone at RT (2.8 × 10^7^ versus 1.3 × 10^7^ plaque-forming units (p.f.u.) per ml), and stabilized the virus >3.4-fold over PBS alone at 37 °C (2.0 × 10^6^ versus 5.8 × 10^5^ p.f.u. per ml). The PEG solution preserved Ad-NSP1 infectivity 10-fold over PBS at RT (1.3 × 10^8^ versus 1.3 × 10^7^ p.f.u. per ml and >48-fold at 37 °C compared with PBS (2.8 × 10^7^ versus 5.8 × 10^5^ p.f.u. per ml) (Supplementary Fig. 10). To determine whether this increase in vector stabilization by sucrose, MUS:OT NPs and PEG impacts the immunogenicity of Ad vectors, mice were vaccinated with 100 μl of the stabilized viruses by intramuscular injection. At 11 days post vaccination, mice were killed and lymphocytes were prepared from the spleen of each animal (*n*=5). Interferon-gamma ELISpot analysis of the splenocytes was used to quantify the frequency of T cells responding to the vaccine (Fig. 4 and Supplementary Fig. 9). Splenocytes were incubated for 2 days in the presence of a NSP1 peptide pool, dimethylsulphoxide (negative control) or phorbol myristate acetate/ionomycin (positive control).

All of the mice had a similar background level of activated T cells (cells treated with dimethylsulphoxide) and similar response to the positive control (phorbol myristate acetate/ionomycin) indicating that stabilization of the Ad vectors had no effect on general T-cell responses in the mice. However, mice vaccinated with Ad-NSP1 stabilized in sucrose had significantly increased frequency of NSP1-specific T cells compared with mice vaccinated with Ad in PBS. This effect was more pronounced for virus stabilized in sucrose at 37 °C compared with RT (T-cell frequency was increased >12 versus 2-fold (Fig. 4)). These data indicate that stabilization with sucrose, at either temperature, markedly increased the functional immunogenicity of Ad-NSP1. It also indicates that there is a threshold of Ad required to elicit T-cell responses and that sucrose stabilization promotes retention of this activity compared with PBS alone. Mice vaccinated with Ad-NSP1 stabilized in PEG at RT had a significant increase in T-cell responses against NSP1 compared with mice vaccinated with Ad-NSP1 stored in PBS. However, mice vaccinated with Ad-NSP1 stabilized in PEG at 37 °C had very similar T-cell responses to mice vaccinated with the Ad-NSP1 stabilized in PBS at 37 °C (Fig. 4). Our *in vivo* results show that we could obtain immunogenicity in mice injected with viral vectors kept at 37 °C for 10 days. These data indicate that Ad stabilization by PEG increased immunogenicity at RT; however, the immunogenicity is diminished when the Ad is stabilized in PEG at 37 °C, despite retention of infectivity indicating that there are additional requirements that are met by stabilization with sucrose but not PEG. Importantly, these results indicate that when our additives show large effects *in vitro*, we observed also a preservation of the *in vivo* immunogenicity of the viral vaccine. The exception are anionic NPs. If they are used for Ad virus stabilization, no difference was found compared with the PBS stored virus despite the good *in vitro* stabilization for the Ad-GFP.

## Discussion

Our study presents simple solutions to the cold chain problem for vaccines, extending the lifetime of virus particles by days and even months at RT and at elevated temperatures. Sugars have the advantages of being inexpensive and have shown the best performance but only at high concentrations. NPs and polyols are more expensive, but show effects at extremely small concentrations. The rate of infectivity loss is characteristic of a degradation mechanism with a single rate-limiting step. This opens up the development of quantitative models of viral degradation mechanisms, a simple but illustrative example of which has been presented in the Supplementary Discussion section. Given the high costs for the development of vaccine formulations, we expect that our results will have an impact especially for the new generations of chimeric vaccines that are currently under development. Moreover, this study could be a starting point to develop more solutions for the stabilization of viral particles, depending on the specific medical need of virus-based applications (that is, vaccines, therapies and viral gene delivery systems).

### Data availability

The data sets generated during and/or analysed during the current study are available from the corresponding authors on reasonable request.

## Additional information

**How to cite this article**: Pelliccia, M. *et al*. Additives for vaccine storage to improve thermal stability of adenoviruses from hours to months. *Nat. Commun.* **7**, 13520 doi: 10.1038/ncomms13520 (2016).

**Publisher's note**: Springer Nature remains neutral with regard to jurisdictional claims in published maps and institutional affiliations.

## Supplementary Material

Supplementary Information Supplementary Figure 1-10, Supplementary Tables 1-10, Supplementary Discussion, Supplementary Methods, and Supplementary References
