# Supplementary material for: Additives for vaccine storage to improve thermal stability of adenoviruses from hours to months
Source: Nat Commun. 2016 Nov 30;7:13520. doi: 10.1038/ncomms13520 (PMC5141364; doi:10.1038/ncomms13520)
Supplement: Supplementary Information — Supplementary Figure 1-10, Supplementary Tables 1-10, Supplementary Discussion, Supplementary Methods, and Supplementary References [file ncomms13520-s1.pdf]

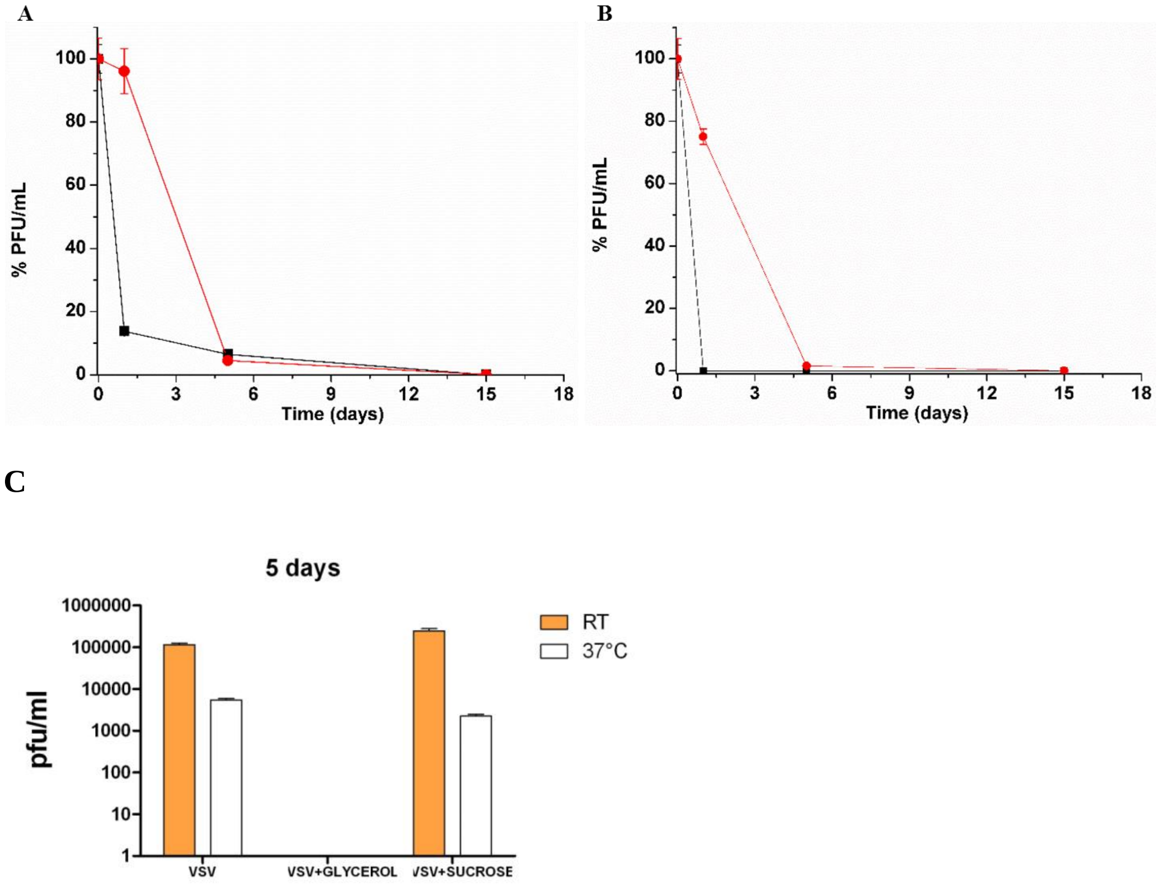

Supplementary Figure 1. Stability of HSV-2 and VSV viruses in medium, glycerol or sucrose solution. Plaque Forming Unit per mL (PFU mL^{-1}) infectivity of HSV-2 virus stored in MEM (black squares) and 0.6 M sucrose (red circle) at RT (A) and (B) at 37°C. (C) Plaque Forming Unit per mL (PFU mL^{-1}) infectivity of VSV stored in glycerol and 0.6 M sucrose after 5 days of storage at RT (orange bars) and at 37°C (white bars). The experiments were performed in duplicate. The error bar present the standard deviation.

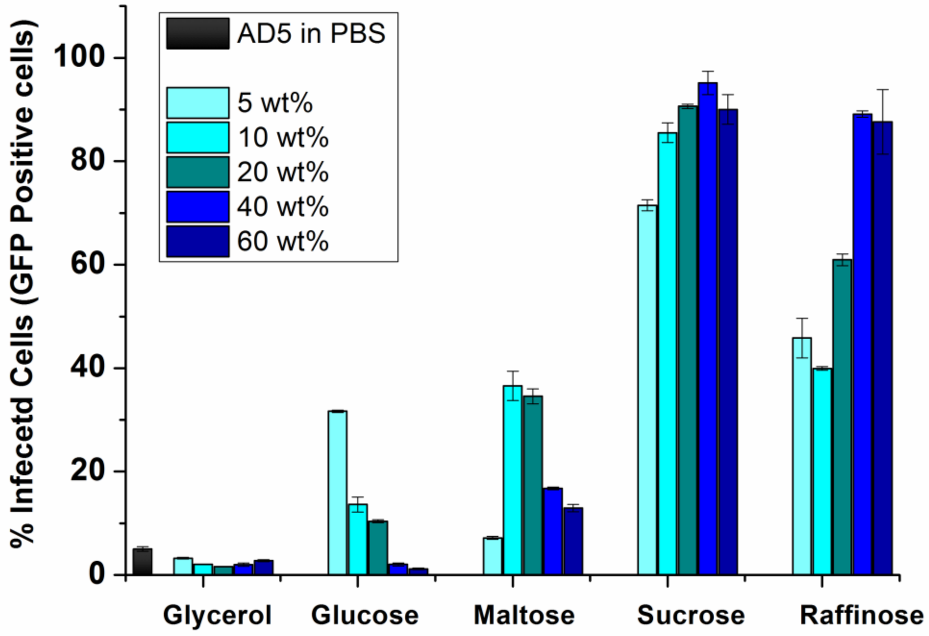

Supplementary Figure 2. Ad5 stability in glycerol and different polysaccharide solutions. Infectivity of Ad5 virus is expressed in % of Infected cells (GFP positive cells) stored for 5 days in different concentrations of molecules as reported in the legend (concentration are expressed in wt%), at 37°C. The experiments were performed in duplicate. The error bar present the standard deviation.

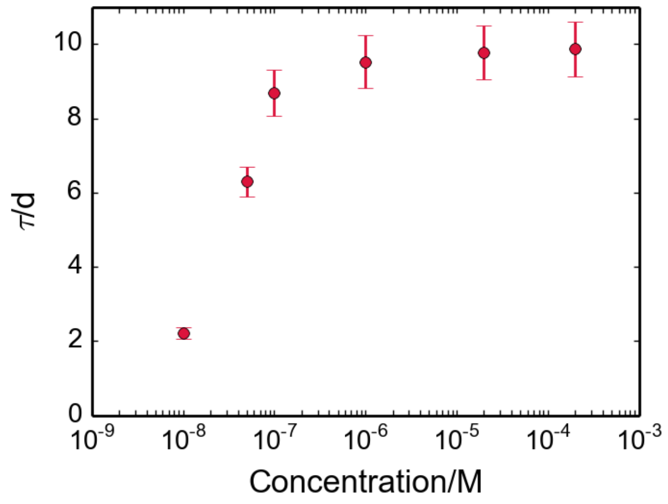

Supplementary Figure 3. Ad5 stability in PEG8000 solutions. Lifetime τ of Ad5 particles as a function of PEG8000 concentration at 37°C, obtained from fits to data shown in Figure 2B in the main text. The experiments were performed in duplicate. The error bar present the standard deviation.

A **B**

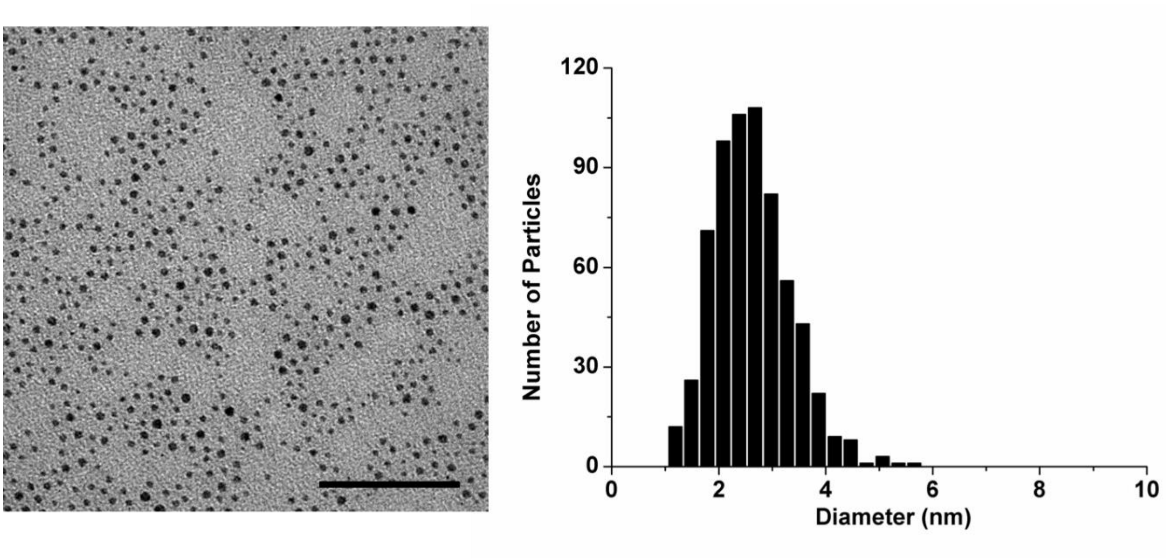

Supplementary Figure 4. Size distribution for the negatively charged MUS:OT NPs. (A) Electron micrographs of the negatively charged MUS:OT coated AuNP. The scale bar indicates 50 nm. (B) Particle size distribution obtained from TEM analysis: the average core diameter was 2.8 nm with a 26% relative standard deviation.

A B

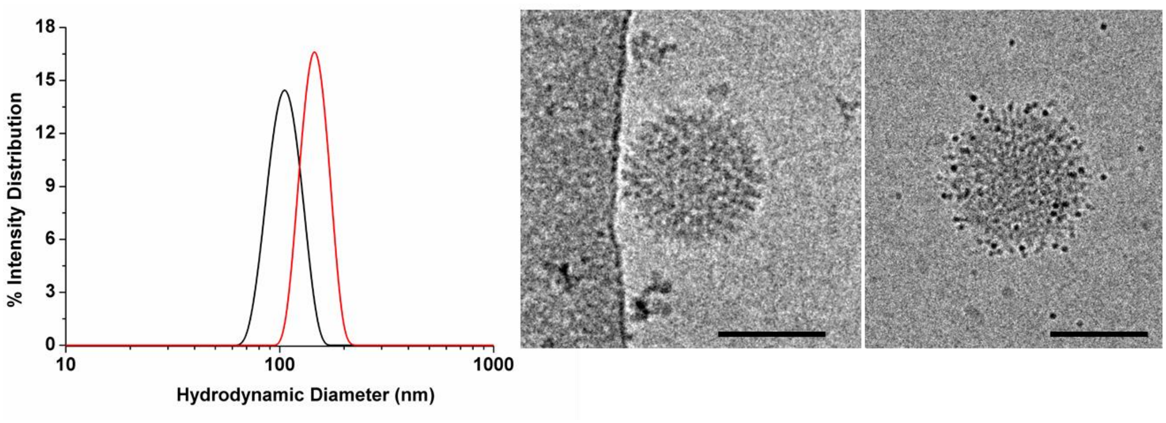

Supplementary Figure 5. Proof of NP-virus interaction. (A) DLS measurement of the intensity (a.u.) based size distribution of Ad5 ($5x10^{10}$ Vp mL^{-1}) at RT (black curve) and for ratio of MUS:OT NP/Ad5~500 after 1 h of incubation at RT (red curve). (B) CRYO-TEM images of the Ad5 alone (left) and Ad5 after 1 hour of incubation with MUS:OT NPs (right). The small dark black dots are the NPs localized close to the viral capsid. Scale Bars indicate 100 nm.

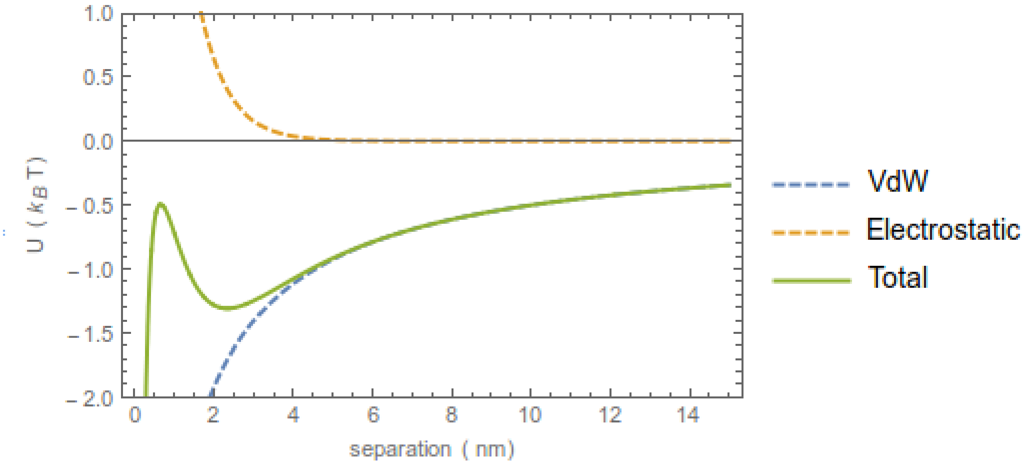

Supplementary Figure 6. Van der Waals, electrostatic, and total interaction energy of negatively charged MUS:OT nanoparticles with Ad5, as a function of separation distance. At intermediate separations, the electrostatic repulsion between the negative surface charges on the nanoparticles and the virus dominates, whereas the van der Waals attraction is stronger at larger separations. The competition between the two interactions gives rise to a potential well of depth roughly -1.3 kT at a separation of roughly 2 nm.

A B

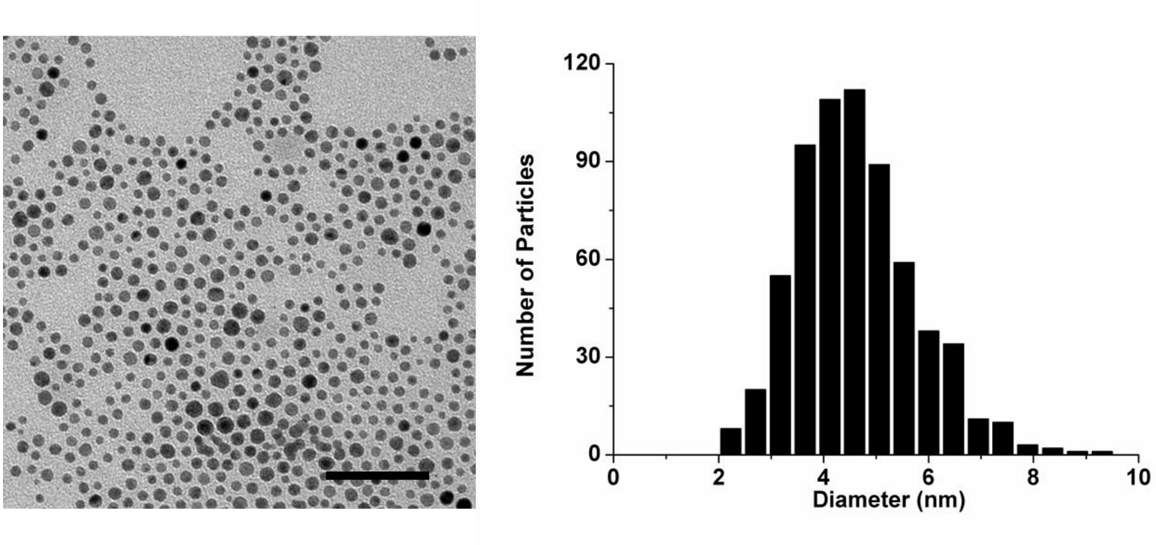

Supplementary Figure 7. Size distribution of the positive TMA:OT NPs. (A) Electron micrographs of the positively charged TMA:OT coated AuNP. The scale bar indicates 50 nm. (B) Particle size distribution obtained from TEM analysis: the average core diameter was 4.9 nm with a 23% relative standard deviation.

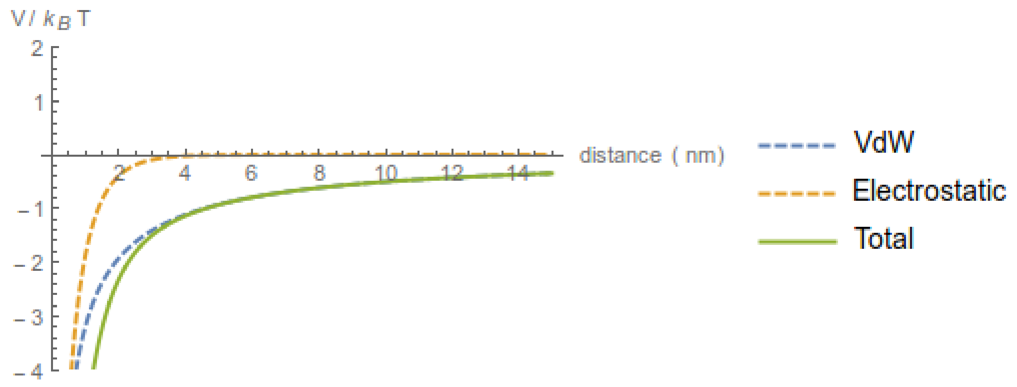

Supplementary Figure 8. Van der Waals, electrostatic, and total interaction energy of positively charged TMA:OT nanoparticles with Ad5, as a function of separation distance. Since the TMA:OT nanoparticles have positive surface charge, the electrostatic contribution is attractive, leading to strong binding between the nanoparticles and the virus.

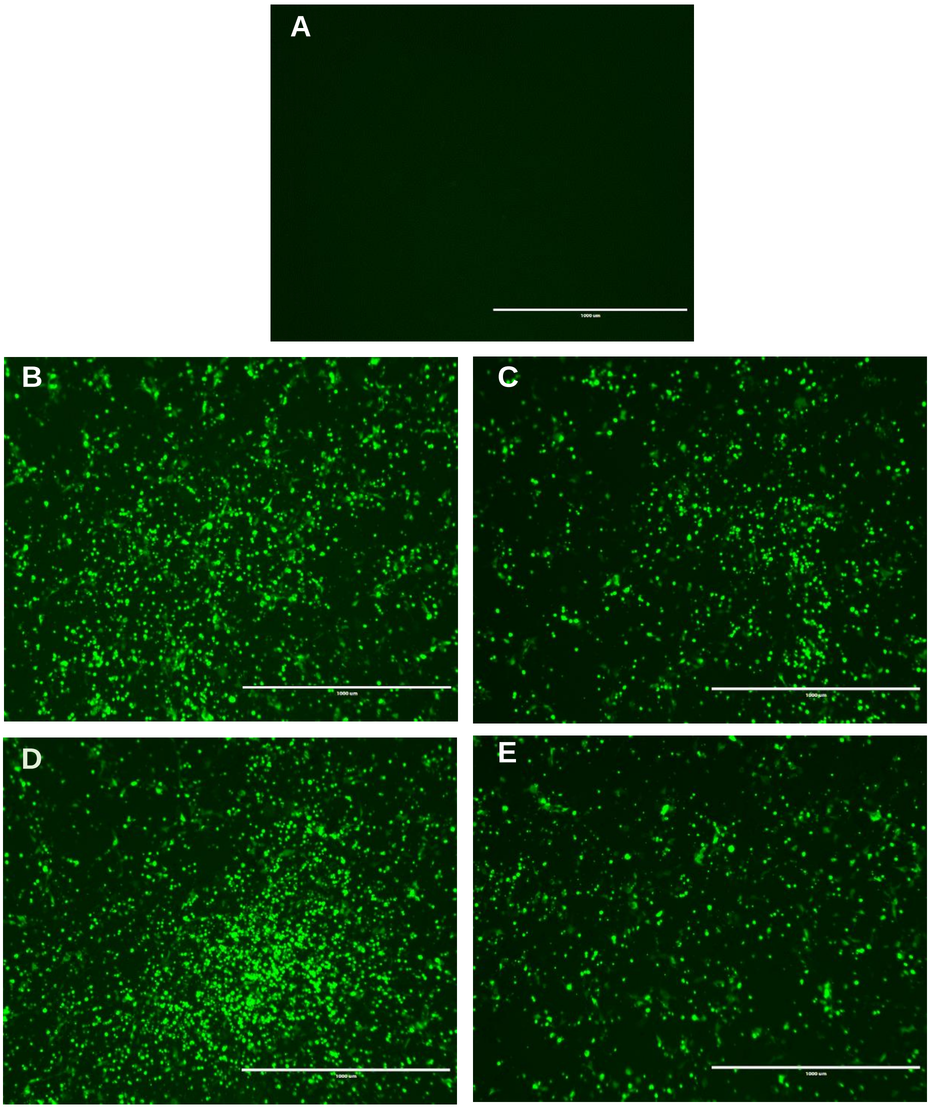

Supplementary Figure 9. Epifluorescence images of GFP positive cells after infection with Ad5 incubated with different negatively charged NPs. Adenovirus stabilization after 5 days at 37°C when incubated with 100 μg mL^{-1} ($1x10^{-7}$ M) of negatively charged NPs of different size and surface composition. All images are fluorescence microscopy images of HEK cells (Human embryonic kidney cells) incubated with the virus/NP medium for 24 h. In the image, the cells infected by an active virus appear as green dots due to GFP expression. (A) virus without NPs; (B) virus with NPs coated with a 2:1 mixture of MUS and 2-7 dimethyl-octyl thiol; (C) virus with NPs coated with a 2:1 mixture of MPSA and OT, here the charged ligand is shorter than the hydrophobic one, (D) NP coated solely with MUS and (E) the 15 nm citrate stabilized gold NP. All the tested negatively charged NPs show a stabilizing potential for 5 days at 37°C. Scale bars are 1000 μm.

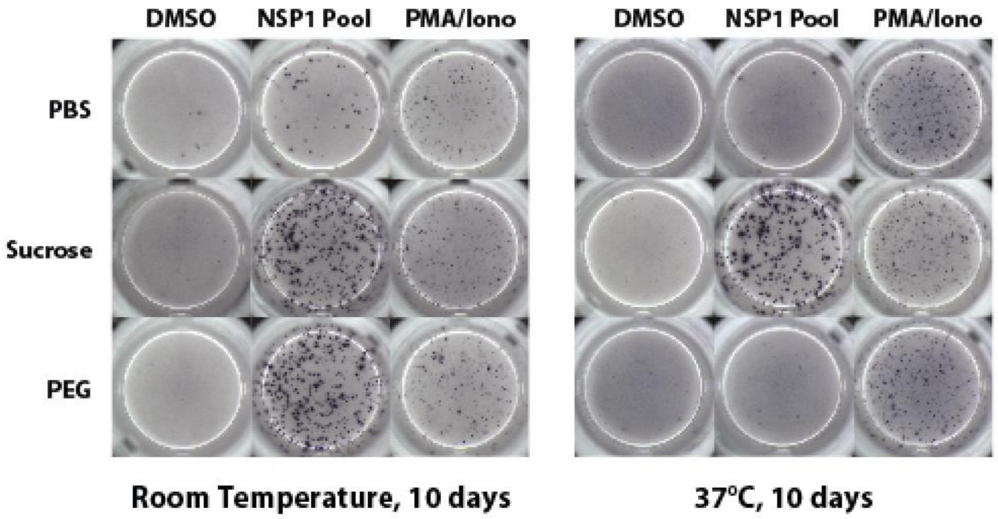

Supplementary Figure 10. Stabilization of AdV-NSP1 enhances immunogenicity. Adenovirus vector expressing NSP1 from Chikungunya virus was incubated for 10 days at room temperature or at 37°C in PBS, 0.6 M Sucrose, or 1 µM PEG8000. Mice were vaccinated by intramuscular injection of 100µl of AdV-NSP1 from each stabilization condition (n=3 per group). At 10 days post vaccination, mouse splenocytes were analysed by IFN-γ ELIspot shown here as photomicrographs of representative wells for the frequency of T cells responding to NSP1 peptides. AdV-NSP1 stabilized in sucrose significantly increased the frequency of T cells responding to NSP1. The analysis of the spots is shown in fig. 4 in the main text.

Supplementary Table 1: S value for PBS

Temperature	S
RT	0.074
37°C	0.025

Supplementary Table 2: S value for Sucrose at 25°C

Concentration	S
0.14 M	0.028
0.29 M	0.024
0.58 M	0.020
1.2 M	0.024
1.7 M	0.024

Supplementary Table 3: S value for Sucrose, 37°C

Concentration	S
0.14 M	0.046
0.29 M	0.023
0.58 M	0.029
1.2 M	0.049
1.7 M	0.073

Supplementary Table 4: S values for MUS:OT at 25°C

Concentration	S
4.5e-09 M	0.029
4.5e-08 M	0.060
1.1e-07 M	0.063
2.2e-07 M	0.070
4.5e-07 M	0.046
1.1e-06 M	0.030
2.2e-06 M	0.022
4.5e-06 M	0.028
9e-06 M	0.046

Supplementary Table 5: S values for MUS:OT at 37°C

Concentration	S

4.5e-09 M	0.017
4.5e-08 M	0.045
1.1e-07 M	0.042
2.2e-07 M	0.037
4.5e-07 M	0.024
1.1e-06 M	0.024
2.2e-06 M	0.022
4.5e-06 M	0.045
9e-06 M	0.043

Supplementary Table 6: S values for TMA:OT NP at a concentration of 1e-7 M

Temperature	S
RT	0.045
37°C	0.051

Supplementary Table 7: S values for PEG at 25°C

Concentration	S
1e-08 M	0.168
5e-08 M	0.372
1e-07 M	0.046
1e-06 M	0.391
2e-05 M	0.053
2e-04 M	0.052

Supplementary Table 8: S values for PEG at 37°C

Concentration	S
1e-08 M	0.059
5e-08 M	0.041
1e-07 M	0.017
1e-06 M	0.035
2e-05 M	0.048
0.0002 M	0.041

Supplementary Table 9: S values for Glucose at 25°C

Concentration	S
0.28 M	0.030
0.56 M	0.036
1.1 M	0.021

2.2 M	0.031
3.3 M	0.021

Supplementary Table 10: S values for Glucose at 37°C

Concentration	S
0.28 M	0.033
0.56 M	0.024
1.1 M	0.022
2.2 M	0.007
3.3 M	0.014

Supplementary Table 11: S values for Glycerol at 25°C

Concentration	S
0.54 M	0.040
1.1 M	0.066
2.2 M	0.117
4.3 M	0.072
6.5 M	0.067

Supplementary Table 12: S values for Glycerol at 37°C

Concentration	S
0.54 M	0.041
1.1 M	0.032
2.2 M	0.019
4.3 M	0.029
6.5 M	0.059

Supplementary Discussion

Model and theoretical estimates

As we described in the main text, the loss of infectivity of Ad5 over time is consistent with a degradation mechanism with a single rate-determining step. Several possibilities exist for the rate-determining step, ranging from denaturation of a key protein in the infection process to premature release of the genome. Our experiments were not designed to definitively identify the rate-determining step. Here, we develop a minimal model of adenovirus degradation assuming a particular rate-determining step, which is that loss of infectivity occurs due to the thermally-induced mechanical rupture of the adenovirus capsid.

Our choice of degradation mechanism is based on two features of adenovirus. First, adenovirus confines a highly negatively charged genome into its capsid at high pressure due the DNA self-repulsion. [1–3] The pressure, measured to be around 30 atm [3], is countered by mechanical stresses in the capsid which may prepare the virion for uncoating during infection. [4] Second, mechanical stresses induce adenovirus disassembly and genome release as demonstrated in atomic force microscopy experiments [5]. As with any microscopic elastic shell [6–8] the capsid experiences stochastic mechanical deformations whose amplitude is set by the temperature and the elastic stiffness. [9] Combined with the pressure-induced background stresses, these random deformations can stress a capsid region past its mechanical breaking point, leading to rupture followed by release of the genome and loss of infectivity.

Internal pressure and elasticity of Adenovirus type 5

Adenovirus type 5 (Ad5) is an icosahedral double-stranded DNA virus that encloses a large genome into a small internal volume. This makes it similar to bacteriophage viruses such as

phage lambda which are known to experience an internal pressure as high as 50 atm due to the close packing of DNA.[1] However, the genome size of Ad5 is smaller than that of phages with similar capsid radius, implying a smaller packing density of DNA within the capsid and hence a lower internal pressure. The internal radius of Ad5 is 31 nm [10] and the strain used in our experiments has a genome roughly 32 kbp long. This corresponds to a packing density slightly less than half that of phage lambda, which packages a 48.5 kbp genome into a 27.5 nm radius capsid.[11] The most estimate of the Ad5 internal pressure comes from Ortega-Esteban et al. [3] which reported pressures of roughly 30 atm based on atomic force microscopy measurements, which is indeed very large yet smaller than the internal pressure of phage lambda, consistent with the lower DNA packing density.

Rupture rate of an ideal spherical shell model of the virus capsid

Adenovirus type 5 has an icosahedral capsid with 20 faces composed of 240 copies of a six-coordinated hexon protein, and twelve five-coordinated pentons (5-sided protein arrangements) at the apices.[12] The capsid is held together by many copies of “glue" proteins that bind specific capsomeres together. The thermal inactivation of Ad5 is driven by the irreversible degradation of the capsid into subunits. The transition temperature associated with this degradation is lower than the subsequent degradation of individual hexon and penton assemblies and the denaturation of the capsid proteins.[13] We model this degradation as a mechanical rupture of the external capsid, which occurs when the elastic strains in some region of the capsid exceeds the maximal strain tolerated by the structure.

In the main text, we identified the primary physical parameters responsible for virus capsid degradation due to cell rupture and derived the scaling of the lifetime set by this mechanical process. Here we derive a mathematical expression for this lifetime in the limit of a perfectly spherical elastic shell permeable to its surrounding fluid, experiencing thermal fluctuations in

its shape. We describe the shell shape in terms of the radial displacement w from the stress-free configuration.

We limit ourselves to analyse the component of w that is uniform over the entire shell (the $l = 0$ component if w is expanded in the usual spherical harmonics) because the enclosed volume of the shell varies linearly with this component compared to quadratically with all the other components; hence the $l = 0$ component couples most strongly to the excess pressure p inside the shell. The analysis could also be carried out for the other deformation modes ($l > 0$); this would change the numerical pre-factors associated with various terms but not the overall scaling dependence of the lifetime on the physical parameters.

Consider a spherical shell with 2D bulk modulus K [related to the 2D Young's modulus Y and Poisson ratio υvia $K = Y/2(1 - \nu)$] and radius R subjected to an internal pressure p. The equation of motion of a shell element of area dA and mass m in the overdamped limit is

$$\alpha \frac{dw}{dt} = -\frac{4K}{R^2} w + p \qquad (1)$$

where α is the drag coefficient that quantifies the friction of the shell with the surrounding fluid, $\alpha \propto \eta$ where η is the fluid viscosity and the proportionality constant depends on geometric parameters. For a porous shell in an incompressible fluid, the drag resisting the l = 0 fluctuations is due to the motion of the viscous fluid through the pores as the shell expands and contracts. Using Darcy's law, a physical estimate of the drag coefficient is $\alpha = 8\eta h/\pi r_0^4\, n$ for pores uniformly distributed over the shell with area density n, characteristic radius r_0 and characteristic length h.[14] This can be written as $\alpha = A\eta/R$ where the dimensionless numerical factor $A \equiv 8hR/\pi r_0^4 n$ encodes the geometric dimensions of the pores and their spatial density over the shell surface. In the work of Kuriabova and Levine [14], A is estimated as 50 for rotavirus, a virus with visible pores 5 nm in diameter. The pores in adenovirus are much smaller, <1 nm, implying a value of A two to three orders of magniture

higher because of the strong dependence on pore size. The pore size was estimated from the x-ray and cryo-EM structures reported in [15,16].

Supplementary Equation 1 describes the dynamics of an individual shell element. The equation needs to be integrated over the whole shell surface to get an equation of motion of the dynamic variable w. However, since our crude model assumes a perfectly uniform shell, the integration amounts to simply multiplying Supplementary Equation 1 with the area of the shell, to get:

$$4\pi R^2 \alpha \frac{dw}{dt} = -16\pi K w + 4\pi R^2 p \equiv -\frac{dE(w)}{dw} \quad (2)$$

where we have defined

$$E(w) = 8\pi K w^2 - 4\pi R^2 p w \quad (3)$$

as the integral of the right-hand side of Supplementary Equation 2 with respect to w. $E(w)$ is interpreted as the net potential energy associated with the deformation of the shell, whose gradient provides the driving force for the dynamical variable w. At zero temperature, the shell attains equilibrium ($\frac{dE}{dw} = 0$) at the displacement value

$$w_0 = \frac{pR^2}{4K} \quad (4)$$

and the energy can also be expressed as

$$E(w) = 8\pi K (w - w_0)^2 + C \quad (5)$$

where $C = -p^2 R^2 \pi / 2K$ is a constant at a given pressure. At finite temperature, however, the shell fluctuates around this equilibrium value. The amplitude of fluctuations is given by the equipartition theorem as

$$\langle (w - w_0)^2 \rangle = \frac{k_B T}{16\pi K}. \quad (6)$$

For a 2D bulk modulus of 1.8 N m^{-1} (calculated from elastic properties measured for a variant of Adenovirus type 5 in Ortega-Esteban et al. [3], Supplementary Equation 6 predicts an RMS fluctuation amplitude of order 0.01 nm. The effect of the shape fluctuations on the dynamics of the w coordinate is modelled by adding a random noise term to the right of Supplementary

Equation 2, whose strength is determined by the temperature and the Darcy coefficient through an Einstein relation. As a result, the dynamic variable w performs a random walk biased by the energy landscape $E(w)$.

We model failure of the shell by introducing a threshold extensional strain u_r that it can withstand. Beyond this rupture strain, the shell breaks. The strain threshold, a property of the shell material, is related to a threshold radial displacement $w_r = Ru_r$. At zero temperature, the shell is stable for all pressures for which $w_0 < w_r$, which gives the rupture pressure

$$p_r = \frac{4Kw_r}{R^2} = \frac{4Ku_r}{R} \qquad (7)$$

at which stability is lost. At finite temperature, the fluctuation shell has a finite probability of attaining the threshold radial displacement even if the pressure is below p_r, because of thermal fluctuations. As the shell shape fluctuates about $w_0(p) < w_r$, it ruptures the moment w first crosses w_r due to a random fluctuation -a classic “escape over a barrier” problem [17,18] for which the rate J_r is set by the equilibrium dynamics of the shell, described by Supplemenatry Equation 2, and the energy barrier $\Delta \mathrm{E} = 8\pi \mathrm{K}(w_r - w_0)^2$.

In the limit of $\Delta E/k_B T \gg 1$, the rate for a kink-shaped energy landscape is

$$J_r = 16\sqrt{\pi}\frac{K}{\gamma}\sqrt{\frac{\Delta \mathrm{E}}{k_B T}}\, e^{\Delta \mathrm{E}/k_B T} \qquad (8)$$

where $\gamma = 4\pi R^2 \alpha = 4\pi A \eta R$ is the drag coefficient.[18] Rather than using the rupture displacement w_r, we may directly represent the energy barrier in terms of the rupture pressure which we assume to be measurable by other methods e.g. osmotic shock. Then we have

$$\Delta E = \frac{\pi}{2}\frac{R^4}{K}(p_r - p)^2 \qquad (9)$$

The mean lifetime τ is the inverse of the rupture rate J_r. Supplementary Equations 8 and 9 describe the rupture dynamics of a uniform spherical shell undergoing thermal fluctuations in its radius. Ignoring numerical prefactors, which would be different for a more realistic model of capsid rupture, we get the scaling forms $\tau_0 \sim \sqrt{\frac{k_B T}{\Delta E}}\frac{\eta R}{K}$ and $\Delta E \sim (p_{in} - p_r)^2 R^4/K$.

Model-based interpretation of adenovirus lifetimes

According to our model, different physical changes to the virus or the surrounding medium may produce dramatically different effects on viral stability. Increasing the viscosity of the medium is expected to produce a modest linear enhancement of virus lifetime. By contrast, additives that increase the strength of capsid protein-protein associations (thus increasing p_r), reduce the pre-stresses due to internal pressure, or otherwise enlarge the energy barrier of rupture, are expected have a dramatic effect on the lifetime. (Note that the square root dependence of τ_0 on ΔE is dominated by the exponential term.) More realistically, the degradation dynamics would be dominated by the capsid strength in weak regions such as the pentons [4,5,19–21]. However, the scaling forms of τ_0 and ΔE would remain valid, with effective elastic properties and rupture strength determined by the local capsid structure in the weak regions.

We now evaluate the enhancement of adenovirus lifetimes in sucrose and other sugars in the context of our hypothetical model. At RT, the virus lifetime in 0.3 M sucrose solution was found to be 30-fold higher than in PBS (main text Fig. 1). Since the viscosity of 0.3 M sucrose is about 30% higher than that of PBS [22], a linear increase in viscosity cannot explain the dramatic improvement in storage lifetime. However, an additional change in ΔE (of roughly 10% relative to PBS at 0.15 M sucrose concentration) can cause the observed improvement in lifetime. For an internal pressure of 30 atm and a bulk modulus of 1.8 N m^{-1}, a 10% increase in ΔE requires an increase in the threshold rupture pressure of less than 1% according to Supplementary Equation 9. Such an increase could potentially be attributed to enhanced protein-protein binding strengths in the capsid (which determines the threshold rupture stress and hence p_r) due to the modification of protein hydration by sucrose. [23] These rough estimates highlight the role of the exponential factor in translating relatively small physical changes into large enhancements in lifetime.

The effect of viscosity on lifetime is relatively small in this particular model. Stabilization experiments using different organic compounds are consistent with this feature, since solutions with similar viscosities to sucrose (glycerol, glucose, maltose) did not induce the same degree of stabilization (Supplementary Fig. 2). The highest stabilizing effect was seen for sucrose and for raffinose, which includes sucrose as a hydrolysis product.

Finally, we comment on the effects of PEG and nanoparticles in the context of our rupture-based model. The striking feature of the newly proposed stabilization additives is their strong effect on adenovirus lifetime at concentrations as low as 10^{-7} M. These concentrations are too low to significantly affect the bulk properties of the medium – in the case of the rupture-based model, the relevant bulk properties are viscosity and osmotic pressure, which are essentially unchanged from PBS. By contrast, the rupture strength (quantified as the rupture pressure p_r) can be changed by specific associations of individual PEG molecules/nanoparticles with the capsid proteins, especially since the rupture strength in the heterogeneous adenovirus capsid is likely determined by protein-protein interactions in specific weak regions (such as the pentons). Therefore, associations between PEG/nanoparticles and proteins at these weak regions would suffice to alter the rupture strength, which would have an exponentially enhanced effect on the lifetime. We have directly observed nanoparticles attached to the adenovirus capsid in cryo-TEM images, as described in the next section.

Nanoparticles with positive and negative surface charges displayed drastically different effects: negatively charged nanoparticles stabilized adenovirus, whereas positively charged nanoparticles were strongly destabilizing. Our estimation of the energy barrier to capsid rupture did not include electrostatic effects. However, our model could be made qualitatively consistent with the distinct behaviours by taking into account the exposure of the negatively charged DNA when the capsid opens up. Electrostatic interactions with positively charged nanoparticles outside the capsid would make DNA exposure thermodynamically favourable, whereas negatively charged nanoparticles would thermodynamically favour confinement of

the DNA within the capsid and suppress capsid rupture. This mechanism has been previously identified as a possible driver in virus self-assembly [24], and our results hint that it could be relevant to viral degradation as well.

In summary, our mechanical model of viral degradation demonstrates how physical considerations of viral degradation mechanisms might be translated into quantitative predictions for virus lifetime in the presence of various additives, thus informing the development of stabilizing agents for viral vector storage. The relevance of our model to adenovirus needs to be tested further by designing appropriate experiments. The key features of the rupture-based model might also apply to other viruses, such as phage viruses, which confine genetic material at high pressure.

Van der Waals-electrostatic interaction between virus and nanoparticles

We consider the effective pair potential between a nanoparticle and a virus particle, including van der Waals and screened Coulomb interactions. The nanoparticle and virus surfaces are both assumed to be uniform spheres.

Van der Waals contribution

The virus is modelled as a uniform protein sphere of radius R_v= 60 nm. The NPs are modeled as a spherical gold core with radius R_{NPs} = 2 nm, surrounded by a protein shell of thickness R_S= 1.5 nm. The van der Waals interaction potential for surface-to-surface separation days is

$$U_{vdw} = \frac{A_{pwp}-A_{pwg}}{6(d+R_S)} \frac{R_{NPs}R_V}{R_{NPs}+R_V} - \frac{A_{pwp}}{6d} \frac{(R_{NPs}+R_S)R_V}{R_{NPs}+R_S+ R_V} \quad (10)$$

where the surface properties are included in the Hamaker constants [25]

- protein surfaces separated by water: $A_{pwp} \approx\ 12 \times 10^{-21}$J [26]

- gold surfaces separated by water: $A_{gwg} \approx 300 \times 10^{-21}$J
- protein and gold surfaces separated by water: $A_{pwg} \approx \sqrt{A_{pwp}A_{gwg}} = 60 \times 10^{-21}$J

Screened Coulomb interaction

The overall surface charge of the Ad5 particles is negative, and described by a zeta potential φ_V= -45 mV. Whereas the van der Waals contribution is always attractive, the screened Coulomb interaction is repulsive for the anionic MUS:OT nanoparticles (zeta potential φ_{NPs} =-35 mV) and attractive for the cationic TMA:OT nanoparticles (φ_{NPs} = +60 mV). The screened Coulomb interaction takes the form[25]

$$U_{sc} = -A\kappa d + A\ln[2\sinh(\kappa d)] - B\ln\tanh\left(\frac{\kappa d}{2}\right), \quad (11)$$

where

$$A = \pi\varepsilon\varepsilon_0({\varphi_{NPs}}^2 + {\varphi_V}^2)\left(\frac{(R_{NPs}+R_s)R_V}{R_{NPs}+R_s+R_V}\right) ; B = \pi\varepsilon\varepsilon_0\varphi_{NPs}\varphi_V\left(\frac{(R_{NPs}+R_s)R_V}{R_{NPs}+R_s+R_V}\right) \quad (12)$$

and $\kappa = 0.7$ nm is the Debye screening length of the medium (phosphate buffer solution). The overall interaction potential is obtained by adding the two contributions from the van der Waals and the electrostatic forces. Supplementary Figures 6 and 8 show the interaction potential as a function of distance from the virus, for MUS:OT (φ_{NPs} =-35 mV): and TMA:OT (φ_{NPs} = +60 mV) coated particles respectively.

Supplementary Methods

In vitro experiments

Viruses

Herpes simplex-2 (HSV-2)

A clinical isolate of HSV-2, kindly provided by Prof. M. Pistello, University of Pisa, Italy, was propagated and titrated by plaque assay on Vero cells. Virus stocks were maintained frozen at -80°C.

Adenovirus5 (Ad5)

Ad5 encoding enhanced green fluorescence protein (Ad-GFP; cat. No. 1060) was purchased from Vector Biolabs (Philadelphia, PA, USA). This recombinant human adenovirus type 5 expresses enhanced green fluorescent protein under the control of a CMV promoter. The titre was $1.5x10^{11}$ Vp mL^{-1} in PBS at 5wt% of Glycerol. Virus was stored at -80°C upon usage. The purity of the virus preparation was confirmed by non-denaturating agarose gel electrophoresis and DLS measurements. The DLS showed a PDI of <0.1 indicative for monodispersity and the Coomassie stained agarose gel showed a single band.

Adenovirus-NSP1 construction and stabilization

The adenovirus vector Ad-NSP1 used for the *in vivo* experiment was constructed using the two-plasmid rescue system AdMaxHi-IQ kit (Microbix) by homologous recombination in 293-IQ cells with pBHGloxE1E3cre (human Ad5 genome lacking E1 and E3 and psi site) and a shuttle plasmid pDC316(io) encoding the CHIKV non-structural protein 1 (NSP1) containing an in-frame Flag tag under an MCMV promoter/lac repressor unit. 293-IQ cells express the *lac* repressor, which inhibits transgene expression, ensuring that recombinant viruses can be reconstituted even in cases wherein the transgene is cytotoxic.

The Ad-NSP1 virus was prepared by propagating virus on 293-IQ cells for 3 days. The supernatant of infected cells was centrifuged for 15 minutes at 3000rpm to pellet cells and cellular debris. The supernatant was collected and spun over a 20% sorbitol cushion at 24,000 rpm for 90 minutes. Following the spin, supernatant was removed and viral pellets were resuspended in PBS, aliquoted, and stored at -80°C.

The virus stock is 2 x10^{11} PFU mL^{-1}. The stock was diluted in PBS and SDS buffer and measured the OD260 titer, which was 5.8x10^{12} Vp mL^{-1}. ASDS-PAGE analysis was performed on 1x10^{10} PFU mL^{-1} AdV-NSP1 using a 4-12% Bis-Tris Protein gel (NuPAGE) under denaturing conditions. The gel was stained with Coomassie brilliant blue to reveal protein bands.

AdV-NSP1 stocks were propagated and titered by limiting dilution assays using 293-IQ cells. Transgene expression was verified by western blotting of lysates from infected mouse fibroblasts not containing the *lac* repressor.

Cell Lines

African green monkey fibroblastoid kidney cells (Vero) (ATCC CCL-81) were grown as monolayers in Eagle’s minimal essential medium (MEM) (Gibco/BRL, Gaithersburg, MD) supplemented with 10% heat inactivated foetal calf serum (FCS) and 1% antibiotic-antimycotic solution (Zell Shield, Minerva Biolabs GmbH, Berlin, Germany).

HeLa cells were maintained in MEM (Minimum Essential Medium) (Gibco®) supplemented with 10% heat inactivated foetal bovine serum (FBS), 1% L-glutamine and 1% Penicillin/Streptomycin.

All the cells were maintained at 37°C under 5% CO_2.

Stabilization assay

For Ad5 virus, 2 μl of virus stock solution (1.5x10^{11} Vp mL^{-1}) was added to the 1 mL of solution containing molecules (Sucrose, Glucose, PEG8000 and Glycerol) at different concentrations for defined time (1, 3, 5, 15, 21, 35, 75 days) and stored at RT and 37°C before infection. As a control, Ad5 was stored in PBS. Then 7 μL of each solution were added to 1

mL of medium in a 12-well flat bottom plate containing 10^5 HeLa cells (MOI=21) for 24-48 h at 37°C with medium supplemented with 2% of FBS. The same experimental conditions were used in presence of MUS:OT (11-mercaptoundecane sulfonate:octanethiol) and TMA:OT (N,N,N-trimethyl(11-mercaptoundecyl) ammonium chloride: octanethiol) NPs. The cells were harvested 24-48 h post-transduction, fixed in 1% paraformaldehyde, stored at 4°C in PBS and protected from light. The fraction of infected cells was quantified by Fluorescence-Activated Cell Sorting (FACS), measuring GFP signal of infected cells. The sample acquisitions were performed with FACScalibur (BD Biosciences; USA) and 10.000 events were acquired for each sample. Untreated cells were set as negative control. Each experiment was performed in triplicate and the data were analysed from two independent experiments

For HSV-2, 10^5 PFU mL^{-1} and a solution containing 0.6 M of sucrose were added to the MEM medium and mixed in a total volume of 100 μL. The virus-compound mixtures were incubated for 0, 1, 5 or 15 days at RT and 37°C, then titrated by plaque assay on Vero cells to determine residual viral infectivity**.** The fraction of infected cells was quantified by Plaque assay method. The cells were infected with serial dilutions of mixtures derived from stabilization assay for 2 h at 37°C. Subsequently the viral inocula were removed from the wells, and the cells were washed with medium twice and overlaid with a medium containing 1.2% methylcellulose (Sigma). After 24 h of incubation at 37°C in 5% CO_2, the supernatant was removed, and the cells were fixed and stained with 0.1% crystal violet in 20% ethanol and viral plaques were counted.

Synthesis of gold nanoparticles

MUS:OT, MPSA:OT, MUS:2-7 dimethyl-octyl thiol(brOT), MUS NP

1.2 mmol of gold salt ($HAuCl_4$) was dissolved in 200 mL of ethanol and 1.2 mmol of the desired thiol ligand mixture (MUS:OT 2:1) was added while stirring the reaction solution,

then a saturated ethanol solution of sodium borohydride ($NaBH_4$) was added drop-wise for 2 h. The solution was stirred for 3 h and the reaction flask was then placed in a refrigerator overnight. The resulting nanoparticles were washed several times (3 to 5) by suspending and centrifuging (5500 rpm) it in methanol, ethanol and then acetone. The product was washed 5 times with DI-water using Amicon® Ultra-15 centrifugal filter devices (10k NMWL). Finally, the particles were suspended in a minimal amount of water and freeze-dried to yield ≈ 250mg of a black powder.

For the quantification of the ligand ratio the Au particles were etched in a solution of 15 mg Iodine (Acros) in 0.6 mL of MeOD-d4 (Sigma) for 30 min under sonication. The spectra revealed 20% OT content, i.e. a 4:1 MUS:OT stoichiometry.

All chemicals were purchased from Sigma Aldrich except for the ligand MUS, which was synthesized following the procedure of Verma *et al* [28]. All solvents purchased were reagent grade and purged with nitrogen gas for more than 30 min prior to the reaction.

The negatively charged MPSA:OT, MUS:2-7 dimethyl-octyl thiol(brOT), MUS NP with a self-assembled layer of sulfonated ligands were prepared following the protocol described for MUS:OT using the according ligand mixtures.

TMA:OT 1:1 NP

A mixture of 50 mL ethanol and 50 mL Toluene was prepared. To three different glass vials, 20 mL aliquots of this mixture were added to completely dissolve the reagents: (i) 247.86 mg (0.5 mmol) of Chloro(triphenylphosphine)gold(I); (ii) 70.48 mg (0.25 mmol) of TMA with 43.4 μL (0.25 mmol) of OT and (iii) 130.45 mg (1.5 mmol) of Borane tert-butylamine complex (reducing agent). Dissolution was completed by sonication for 15 min. The gold-salt solution was added to a 250 mL the round-bottom flask with an additional 40 mL of the solvent mixture, followed by addition of the TMA:OT (1:1) mixture. The solution was vigorously (800 rpm) stirred for 10 minutes at room temperature. The reducing agent was then added and the flask was connected to a condenser. The flask containing all the reagents was

directly put into an oil heating bath at 112°C and left stirring at 800 rpm for 1.5 h. The reaction flask was taken out of the heating bath and cooled under stirring (800 rpm) for another hour. To precipitate the product, excess toluene was added, followed by evaporation of the ethanol in a rotary evaporator. Once a black precipitate was visible, the evaporation could be stopped, and the suspension was transferred to 50 mL Falcon™ Tubes, in which the product was washed several times by successive suspension-pelleting cycles (5500 rpm) using acetone. After six washes, the product was dried *in vacuo* to remove the acetone, then dissolved in approximately 30 mL of Milli-Q grade water, and washed 5 times using Amicon® Ultra-15 centrifugal filter devices (30k NMWL). NMR measurements confirmed that the ratio of the ligands was 1:1 within the error range.

All chemicals and solvents were purchased from Sigma Aldrich except for the ligand TMA, which was synthesized following a modification of the protocol of Verma et al.[28]

Citrate Au NP

Monodisperse gold citrate NPs were prepared by a modified protocol from Turkevich et al.[29]. For nanoparticles with size of 18 ± 2 nm, 5.3 mg of $NaAuCl_4 * 2H_2O$ in 25 mL of Milli-Q grade water were mixed at RT with 1 mL of a 1% trisodium citrate solution. Then glass beads were added and the mixture was microwave-irradiated for 90 s. Finally, the deep-red solution was cooled down to RT and stored protected from light until use.

Calculation of molecular weight of gold nanoparticles

The calculations of the molecular weight of the NPs followed the equations described in [30]. NP are composed of a gold core and a monolayer of organic molecules arranged onto NPs surface. A reasonable estimation of the molecular weight of NP is made by following equations:

$$M_{tot} = M_{core} + M_{shell} \qquad (13)$$

$$V_{tot} = V_{core} + V_{shell} \quad (14)$$

$$V_{tot} = 4/3\,\pi r_c^3 + 4/3\,\pi r_{shell}^3 \quad (15)$$

$$M_{tot} = \left(\rho_{Au} V_p + \rho_{ligand} V_{shell}\right) \quad (16)$$

$$MW = M_{tot} \times N_A \quad (17)$$

where N_A is the Avogadro's number, the density of gold bulk, ρ_{Au} is 19.32 gcm^{-3} and the density of the ligand, ρ_{ligand} is 1.2 gcm^{-3}.[30] The length of MUS and TMA is approximately 1.7 nm, the radius of MUS:OT NP, r_c is 1.4 nm. The molecular weight, MW of MUS:OT NP is $2.2\text{x}10^5$ Da. The number of NP in 1 mg is $3\text{x}10^{15}$. In case of TMA:OT the MW is $9\text{x}10^5$ Da, 1 mg of TMA:OT contains $5\text{x}10^{14}$ NP.

Dynamic Light Scattering (DLS) and ζ-potential

A Zeta Nanosizer unit, Malvern NZ (Malvern, UK), was used to perform dynamic light scattering and zeta potential measurements, with laser at 633 nm wavelength. The instrument was operating in back scattering mode (BSM) at an angle of 173°. Measurements were run at least 5.0 min (5 measurements for each sample, 25 runs, 5 seconds each). The measurements were performed at 25°C. Nanoparticle solution in water and PBS were sonicated 15 min at room temperature and filtered 3 times with filter having 0.22 μm pore size before measurements. High concentration zeta cell kit was used to perform ζ-potential measurements in PBS.

The suspensions of Ad5 and NPs was prepared keeping the Ad5-particles number density fixed ρ_{Ad_5} to $5\text{x}10^{10}$ mL^{-1} and MUS:OT NPs number density $\rho_{MUS:OT} = \rho_{Ad_5}(N_{MUS:OT}/N_{Ad5})$, with ratio $N_{MUS:OT}/N_{Ad5} \sim 500$. The mixture was prepared by adding drop-wise 0.5 mL of MUS:OT NPs dispersion to the 0.5 mL of Ad5 viral particle in PBS.

Cryo-Electron Microscope (cryo-EM)

Samples for cryo transmission EM were frozen with a Leica EM GP immersion freezer (Leica Microsystems, Vienna, Austria) following the protocol of Resch et al. [31]. The instrument's environmental chamber operated at 37°C and 95% relative humidity. 10^9 Ad5 particles were incubated with 10^{13} of MUS:OT, NPs for 1 h at 37°C. Each specimen was applied onto a glow discharged 300 mesh copper (Cu) grid coated with perforated Quantifoil R1.2/1.3 carbon films (Quantifoil, Jena, Germany).[32] After 30s settling time, the suspension was blotted for 0.9s with Whatman No. 1 filter paper using the instrument's blotting sensor and immediately plunged into liquid ethane at 89.15K. The vitrified specimens were visualized at liquid nitrogen temperature on a Tecnai F30 Helium (Polara) cryo-TEM (FEI Company, Eindhoven, Netherlands) operated at 300 kV. Low dose micrographs were acquired at nominal magnification of 59000x, at an underfocus suitable for high contrast, and captured with a Gatan US4000 CCD camera.

***In vivo* experiments**

Adenovirus-NSP1 stabilization

For stabilization experiments, 5µL of virus stock solution was added to 1 mL of stabilization solution containing 0.6M sucrose in phosphate buffered saline (PBS) or PBS alone to a final concentration of $1x10^8$ PFU mL^{-1}. The $1x10^8$ PFU mL^{-1} concentration of Ad-NSP1 vector was chosen specifically for the in vivo experiments. In mice, $1x10^7$ PFU per 100µL is a sufficient concentration to induce T cell responses, but below this concentration, T cell responses tend to wane. The solutions were kept at room temperature or at 37°C for 10 days. Effects of stabilization were determined by 1) quantifying AdV-NSP1 titer by limiting dilution and 2) assessing *in vivo* vaccine efficacy.

Mouse model

Female C57Bl/6J mice were obtained from Jackson Laboratories and housed at Oregon Health & Science University in accordance with the NRC guide for the Care and Use of Laboratory Animals and the Animal Welfare Act. Mice were vaccinated by intramuscular injection of 100µl of stabilized AdV-NSP1 virus (n=5). Mice were euthanized at 11 days post vaccination and spleens were collected. Spleen tissues were macerated through a 70µm nylon cell strainer (BD Biosciences) and the washed splenocyte suspension was treated with 0.83% ammonium chloride for 3 minutes at room temperature to promote red blood cell lysis. This process was stopped by the addition of 10ml of Rosewell Park Memorial Institute (RPMI) 1640 medium supplemented with 10% foetal bovine serum (FBS) and penicillin, streptomycin, and glutamine (RPMI-10). Splenocytes were pelleted and resuspended in RPMI-10.

Chikungunya NSP1 peptide library

The CHIKV NSP1 peptide library, purchased from Thermo Fisher Scientific, contains 18-mer peptides that overlap by 12 amino acids spanning the entire NSP1 open reading frame. Peptides were resuspended to 1mg mL^{-1} in DMSO and combined at an equal concentration to produce the NSP1 pool.

ELISpot Assays

Interferon gamma (IFN-γ) positive T cells from the vaccinated animals were detected using the Mouse IFN-γ ELISpot^{PLUS} kit (Mabtech). IFN-γ monoclonal antibody AN18 pre-coated ELISpot plates were washed 4 times with 200µl per well PBS and blocked for two hours in RPMI-10 as per manufacturer's instructions. After removal of the blocking solution, stimuli were added to the appropriate wells and included: 1) 1µg of NSP1 pool; 2) 1µl of a 1:3

dilution of the Leukocyte activation cocktail (BD Biosciences); or 3) 1µl of DMSO. Lymphocyte counts for each splenocyte preparation were obtained using a Hemavet HV950 FS Multispecies Hematologic Analyzer (Drew Scientific) and 2x10^5 lymphocytes in RPMI-10 were added per well. Plates were incubated undisturbed at 37°C for 48 hours. Following incubation, plates were washed and then incubated with primary monoclonal antibody R4-6A2 at 1µg mL^{-1} in PBS with 0.5% FBS for 2 hours at RT. Plates were then washed 5 times with PBS, and spot-formation was visualized using a secondary antibody conjugated to streptavidin-ALP followed by the addition of filtered substrate solution (BCIP/NBT-plus). When distinct spots emerged, the reaction was stopped by rinsing the plates with water. Dried plates were read with an AID ELISpot plate reader.

Statistical analysis

Log-transformed data were analysed by Two-way ANOVA followed by multiple comparison analysis (Dunnett's test) using Prism Graph Pad software, no post-test corrections were carried out due to the small sample size.

Model and quality-of-fit statistics for fraction of cells infected

The cell infectivity curves are fit against the expected theoretical form, Equation 1 in the main text, using a nonlinear least-squares regression with m_0 and τ as fit parameters.[27] When different concentrations of a storage medium were tested concurrently at a particular temperature, a common value of m_0 was assumed among all concentrations. The parameters are reported along with their estimated standard error from the fit.

To judge the effectiveness of the fits in describing the infectivity curves, we calculated the standard error of the estimate, S, for each curve fit to N points:

$$S = \sqrt{\frac{\sum_{i=1}^{N}(y_i - f(x_i))}{N}} \qquad (13)$$

where y_i and $f(x_i)$ are respectively the measured infectivity and the predicted infectivity from the fit at the *i*th data point. *S* measures the typical deviation of the measured infectivity from the best-fit curve, in units of probability. The values of *S* for each infectivity curve are reported in the Supplementary tables 1-12.

Typical values are ≤0.04, i.e. the model curve is within four percentage points of the measured infectivity on average. However, the fit performs poorly for a few storage media at 25°C. In many cases, this is because there is insufficient drop in the infectivity over 21 days for the fit parameters to be precisely determined.

Supplementary References

1. Gelbart, W. M. & Knobler, C. M. Virology. Pressurized viruses. *Science* **323,** 1682–3 (2009).
2. Šiber, A., Božič, A. L. & Podgornik, R. Energies and pressures in viruses: contribution of nonspecific electrostatic interactions. *Phys Chem Chem Phys.* **14,** 3746–65 (2012).
3. Ortega-Esteban, A. *et al.* Mechanics of Viral Chromatin Reveals the Pressurization of Human Adenovirus. *ACS Nano* **9,** 10826–10833 (2015).
4. Pérez-Berná, A. J. *et al.* The role of capsid maturation on adenovirus priming for sequential uncoating. *J. Biol. Chem.* **287,** 31582–95 (2012).
5. Ortega-Esteban, A. *et al.* Monitoring dynamics of human adenovirus disassembly induced by mechanical fatigue. *Sci. Rep.* **3,** 1434 (2013).
6. Park, Y. *et al.* Measurement of red blood cell mechanics during morphological changes. *Proc. Natl. Acad. Sci.* **107,** 6731–6736 (2010).
7. Paulose, J., Vliegenthart, G. A., Gompper, G. & Nelson, D. R. Fluctuating shells under pressure. *Proc. Natl. Acad. Sci.* **109,** 19551–19556 (2012).
8. *Statistical Mechanics of Membranes and Surfaces, 2nd Edition, World Scientific 2004.* (World Scientific, 2004).
9. May, E. R. & Brooks, C. L. Determination of Viral Capsid Elastic Properties from Equilibrium Thermal Fluctuations. *Phys. Rev. Lett.* **106,** 188101 (2011).
10. Carrillo-Tripp, M. *et al.* VIPERdb2: An enhanced and web API enabled relational database for structural virology. *Nucleic Acids Res.* **37,** (2009).
11. Evilevitch, A. & Castelnovo, M. in *Emerging topics in physical virology* (eds. Stockley, P. & Twarock, R.) 217–253 (2010). doi:10.1142/p673
12. Russell, W. C. Adenoviruses: Update on structure and function. *Journal of General Virology* **90,** 1–20 (2009).
13. Ihnat, P. M. *et al.* Comparative thermal stabilities of recombinant adenoviruses and hexon protein. *Biochim. Biophys. Acta - Gen. Subj.* **1726,** 138–151 (2005).
14. Kuriabova, T. & Levine, A. J. Nanorheology of viscoelastic shells: Applications to viral capsids. *Phys. Rev. E - Stat. Nonlinear, Soft Matter Phys.* **77,** 031921 (2008).
15. Liu, H. *et al.* Atomic structure of human adenovirus by cryo-EM reveals interactions among protein networks. *Science* **329,** 1038–1043 (2010).
16. Reddy, V. S., Natchiar, S. K., Stewart, P. L. & Nemerow, G. R. Crystal structure of human adenovirus at 3.5 A resolution. *Science* **329,** 1071–1075 (2010).
17. Zhou, H.-X. Rate theories for biologists. *Q. Rev. Biophys.* **43,** 219–293 (2010).
18. Kramers, H. A. Brownian motion in a field of force and the diffusion model of chemical reactions. *Physica* **7,** 284–304 (1940).
19. Rexroad, J. *et al.* Structural stability of adenovirus type 5. *J. Pharm. Sci.* **92,** 665–678 (2003).
20. Rexroad, J., Evans, R. K. & Middaugh, C. R. Effect of pH and ionic strength on the physical stability of adenovirus type 5. *J. Pharm. Sci.* **95,** 237–247 (2006).
21. Reddy, V. S. & Nemerow, G. R. Structures and organization of adenovirus cement proteins provide insights into the role of capsid maturation in virus entry and infection. *Proc. Natl. Acad. Sci. U. S. A.* **111,** 11715–20 (2014).
22. Swindells, J. F. & States., U. Viscosities of sucrose solutions at various temperatures: tables of recalculated values. 7 p. (1958). at <file://catalog.hathitrust.org/Record/007290919>
23. Timasheff, S. N. The control of protein stability and association by weak interactions with water: how do solvents affect these processes? *Annu. Rev. Biophys. Biomol.*

Struct. **22,** 67–97 (1993).
24. Ting, C. L., Wu, J. & Wang, Z.-G. Thermodynamic basis for the genome to capsid charge relationship in viral encapsidation. *Proceedings of the National Academy of Sciences* **108,** 16986–16991 (2011).
25. Khan, A. A. *et al.* pH Control of the Electrostatic Binding of Gold and Iron Oxide Nanoparticles to Tobacco Mosaic Virus. *Langmuir* **29,** 2094–2098 (2013).
26. Roth, C. M., Neal, B. L. & Lenhoff, A. M. . Van der Waals interactions involving proteins. *Biophys. J.* **70,** 977–987 (1996).
27. Newville, M., Ingargiola, A., Stensitzki, T. & Allen, D. B. LMFIT: Non-Linear Least-Square Minimization and Curve-Fitting for Python¶. Zenodo. 10.5281/zenodo.11813 (2014). doi:10.5281/zenodo.11813
28. Verma, A. *et al.* Surface-structure-regulated cell-membrane penetration by monolayer-protected nanoparticles. *Nat. Mater.* **7,** 588–595 (2008).
29. Turkevich, J., Stevenson, P. C. & Hillier, J. A study of the nucleation and growth processes in the synthesis of colloidal gold". *Discuss. Faraday. Soc.* **11,** 55–75 (1951).
30. Carney, R. P. *et al.* Determination of nanoparticle size distribution together with density or molecular weight by 2D analytical ultracentrifugation. *Nat. Commun.* **2,** 335 (2011).
31. Resch, G. P. *et al.* Immersion freezing of biological specimens: Rationale, principles, and instrumentation. *Cold Spring Harb. Protoc.* **6,** 778–782 (2011).
32. Aebi, U. & Pollard, T. D. A glow discharge unit to render electron microscope grids and other surfaces hydrophilic. *J. Electron Microsc. Tech.* **7,** 29–33 (1987).
